# The Coagulation Factors Fibrinogen, Thrombin, and Factor XII in Inflammatory Disorders—A Systematic Review

**DOI:** 10.3389/fimmu.2018.01731

**Published:** 2018-07-26

**Authors:** Kerstin Göbel, Susann Eichler, Heinz Wiendl, Triantafyllos Chavakis, Christoph Kleinschnitz, Sven G. Meuth

**Affiliations:** ^1^Department of Neurology with Institute of Translational Neurology, University of Münster, Münster, Germany; ^2^Department of Clinical Pathobiochemistry, Laboratory Medicine, Institute for Clinical Chemistry, University Clinic Carl Gustav Carus, Technische Universität Dresden, Dresden, Germany; ^3^Department of Neurology, University Hospital Essen, Essen, Germany

**Keywords:** coagulation factors, neuroinflammation, contact system, fibrinogen, factor XII, thrombin

## Abstract

**Background:**

The interaction of coagulation factors has been shown to go beyond their traditional roles in hemostasis and to affect the development of inflammatory diseases. Key molecular players, such as fibrinogen, thrombin, or factor XII have been mechanistically and epidemiologically linked to inflammatory disorders like multiple sclerosis (MS), rheumatoid arthritis (RA), and colitis.

**Objectives:**

To systematically review the evidence for a role of coagulation factors, especially factor XII, fibrinogen, and thrombin in inflammatory disorders like MS, RA, and bowel disorders.

**Methods:**

A systematic literature search was done in the PubMed database to identify studies about coagulation factors in inflammatory diseases. Original articles and reviews investigating the role of the kallikrein–kinin and the coagulation system in mouse and humans were included.

**Results:**

We identified 43 animal studies dealing with inflammatory disorders and factors of the kallikrein–kinin or the coagulation system. Different immunological influences are described and novel molecular mechanisms linking coagulation and inflammation are reported.

**Conclusion:**

A number of studies have highlighted coagulation factors to tip the balance between hemostasis and thrombosis and between protection from infection and extensive inflammation. To optimize the treatment of chronic inflammatory disorders by these factors, further studies are necessary.

## Introduction

The coagulation system is a highly regulated cascade that ultimately leads to blood clot formation. The primary purpose of coagulation is hemostasis, i.e., to stop bleeding from a damaged blood vessel.

The concept of a stepwise process or cascade of the coagulation system was first described in 1964 (1). While this traditional model described two separate pathways, the intrinsic and the extrinsic pathway, which culminate in a common pathway, current views support an interconnected relationship between the two (2, 3). The contact pathway (also called the kallikrein–kinin system) is composed of three zymogens [factor XII (FXII), plasma kallikrein (PK), and high-molecular-weight kininogen (HMWK)]. *In vitro*, the initial triggering event leads to clot formation through the activation of FXII on an artificial surface. However, *in vivo*, the activation of this factor is still under discussion, so that current studies consider tissue factor (TF), a transmembrane glycoprotein expressed in perivascular tissue, to be the main initiator of *in vivo* blood clotting (4). TF forms a complex with factor VII (FVII) to activate factor X (FX) either directly or by activating factor IX (FIX). Both pathways interlace with the activation of FX, which leads to the cleavage of prothrombin (factor II) to thrombin (factor IIa). In the last step, thrombin mediates the cleavage of fibrinogen to fibrin monomers that, upon polymerization, form a fibrin clot and stop bleeding. The formation of these clots is dependent on the availability of thrombin, calcium, and negatively charged phospholipid membranes. The whole coagulation cascade is very tightly regulated with several checkpoints that function in a positive or negative feedback loop (5).

However, in recent years, significant evidence has emerged implicating coagulation factors also in tissue repair and inflammatory responses. In line with this, several of the major coagulation factors, like TF, thrombin, or fibrinogen, are described as potential drivers of inflammation in disease models, such as sepsis, endotoxemia, encephalomyelitis, or multiple sclerosis (MS) (2, 6–8). Thereby, these factors not only enhance inflammation in the bloodstream, but also within tissues. Furthermore, it is known that FXII triggers the release of bradykinin (BK) from HMWK through cleavage by PK, leaving two chain HMWK behind, which has numerous adhesion-regulatory properties (9, 10) including inhibitory activity on the interaction between fibrinogen and the leukocyte integrin CD11b/CD18 (11). Binding of BK to the BK receptors can activate proinflammatory pathways that induce chemotaxis of leukocytes and increase vascular permeability (12).

A proteomic analysis has been performed on human brain material from individuals with MS identifying a dysregulation of several proteins of the coagulation cascade, such as TF or protein C inhibitor (13). Furthermore, in an animal model of MS, i.e., experimental autoimmune encephalomyelitis (EAE), it could be shown that other factors, such as FXII or thrombin, are upregulated in the central nervous system (CNS) (14, 15). It has been demonstrated that this dysregulation of the coagulation system is not restricted to the CNS but can also be found in the peripheral blood (15). Both FXII and thrombin are highly upregulated in the plasma of patients with MS (16). Moreover, dysregulation of BK receptors was found to be relevant in MS (17, 18).

Although further studies using animal models of MS are required, the available data indicate that the interplay between coagulation factors and immune cells and/or brain endothelial cells may modulate initiation and/or the course of neuroinflammatory disorders.

In this review, we summarize key links between inflammation and coagulation, with a specific focus on the molecular roles of the clotting factors FXII, fibrinogen, and thrombin in neuroinflammation as well as in neuroinflammatory disorders. The role of coagulation factors in non-neurological inflammatory disorders is also discussed. The evidence presented here suggests that manipulation of components of the coagulation system could be potentially therapeutically exploitable not only in inflammatory disorders of the CNS but also in autoimmune diseases in general.

## Methods

A literature review was done in December 2017 searching the PubMed database using the search items: BK, coagulation factors, colitis, complement, Crohn’s disease, EAE, FXII, fibrinogen, inflammatory bowel disease, kallikrein–kinin system, thrombin, MS, and rheumatoid arthritis (RA). The search terms were used in different combinations and plural forms, and the search was limited to articles in English. References were screened for additional articles. Studies in mouse and human were included.

## Results and Discussion

### Factor XII and Neuroinflammation

**Figure 1:**
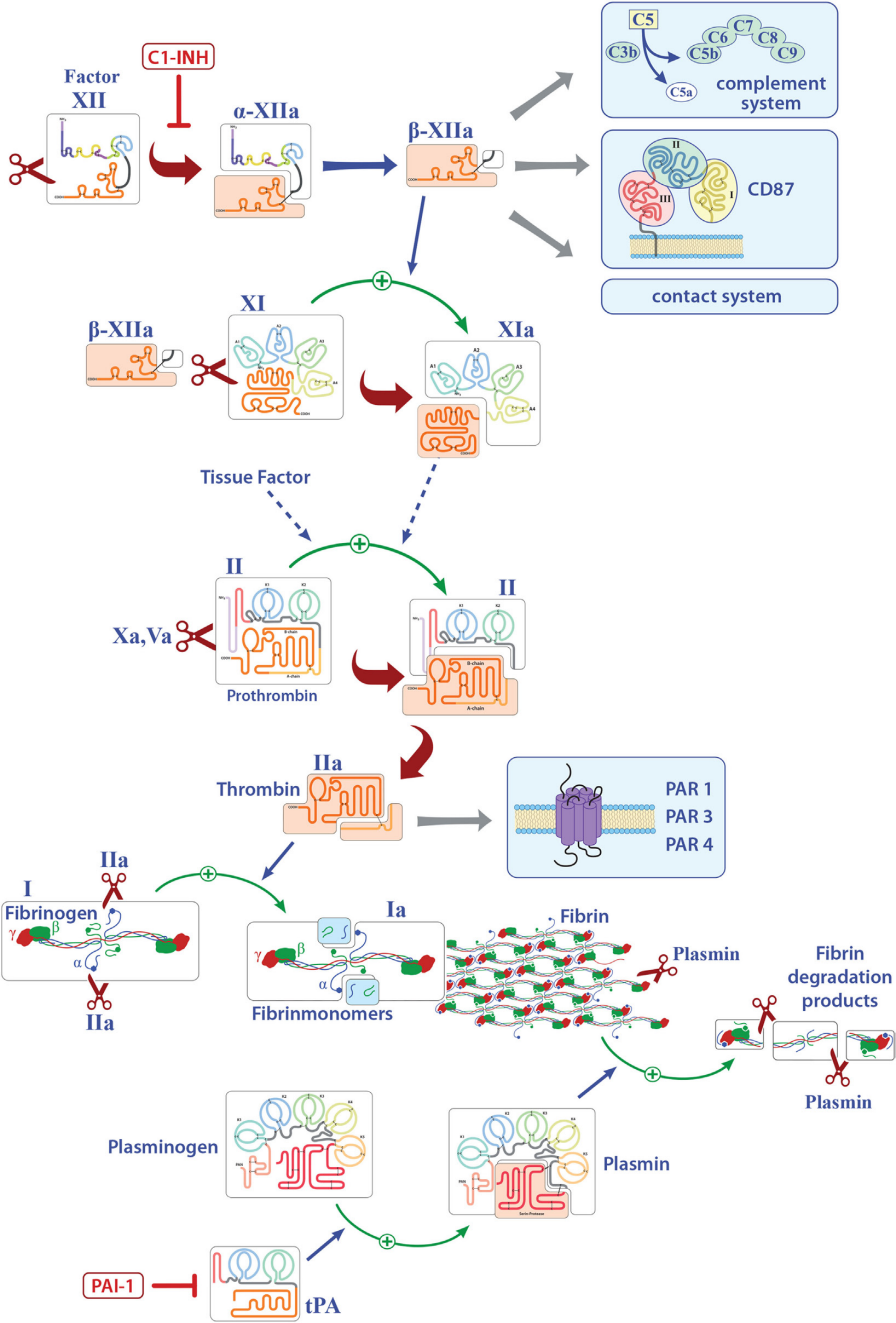
Overview of the known pathways activated by factor XII (FXII) and the extrinsic coagulation system. Only factors that are involved in inflammation are shown. Activated FXII leads to the cleavage of factor XI, activates the intrinsic, the contact, and complement systems and can bind to CD87. Tissue factor finally leads to the release of thrombin (FIIa) that can directly bind several receptors and activates fibrinogen to fibrin. Deposition of fibrin is regulated by plasmin. Abbreviations: C1-INH, serine protease C1 inhibitor; CD87, urokinase-type plasminogen-activator receptor; tPA, tissue plasminogen activator; PAI-1, plasminogen-activator inhibitor 1; PAR, protease-activated receptor.

**Figure 2:**
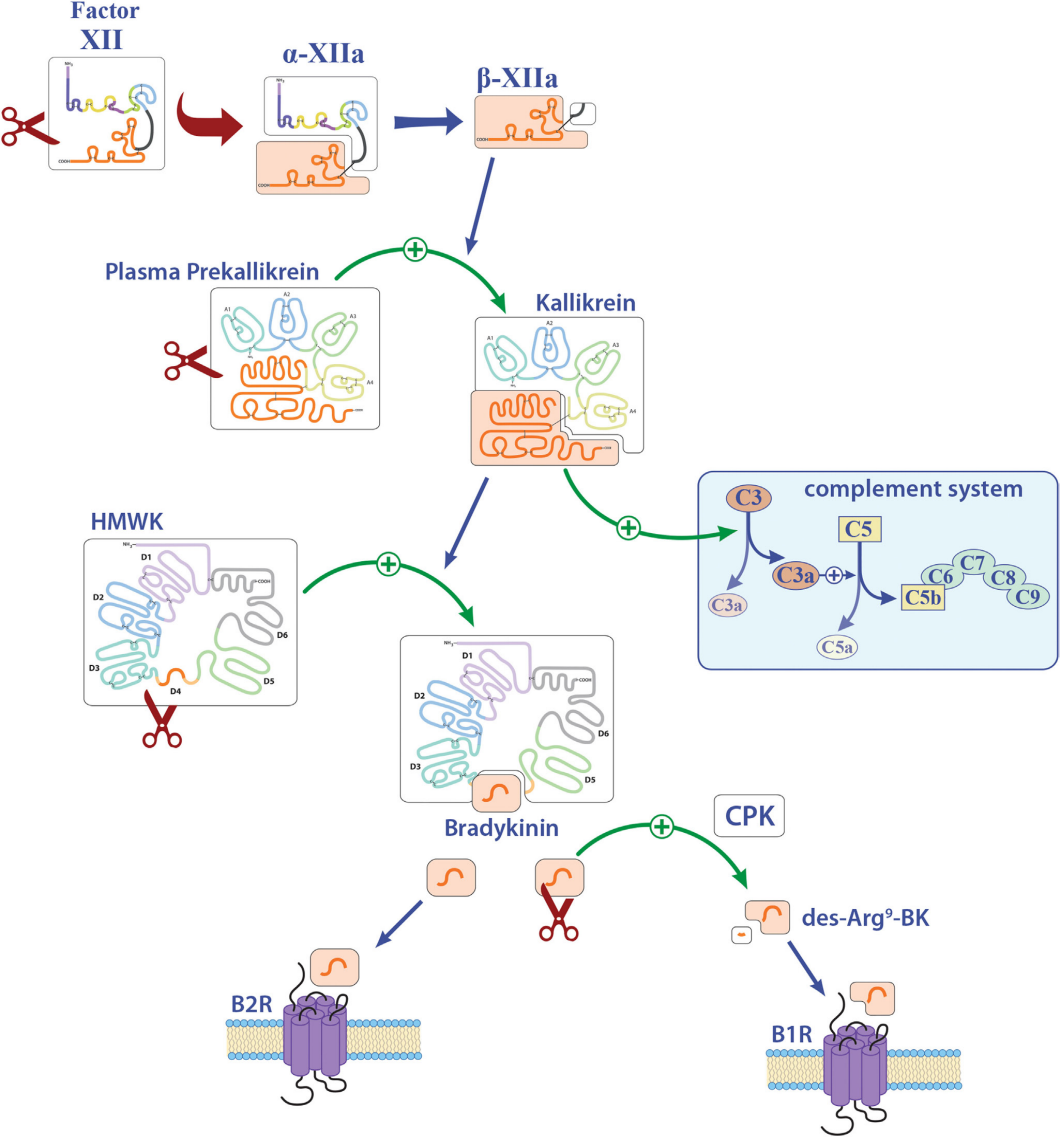
Overview of the contact system. Activation of factor XII (FXII) leads to the cleavage of plasma prekallikrein to kallikrein. Kallikrein can activate both the complement factors C3 and C5 and also high-molecular-weight kininogen (HMWK). Abbreviations: B1R, bradykinin 1 receptor; B2R, bradykinin 2 receptor; BK, bradykinin; CPK, creatinine phosphokinase.

Factor XII is a soluble zymogen with a molecular weight of approximately 80 kDa that is produced in the liver (3). FXII consists of a heavy chain (353 residues) and a light chain (243 residues) held together by a disulfide bond (Figure 1) (19). It has several domains, namely, a leader peptide, a fibronectin type II domain, an epidermal growth factor (EGF)-like domain, a fibronectin type I domain, a second EGF-like domain, a kringle domain, a proline-rich region, and the catalytic domain (Figure 1). Proteolytic cleavage of its R353–V354 site converts the zymogen FXII to activated FXII (FXIIa). This cleaved protein circulates as a two-chain protein, a heavy and a light chain, held together by a disulfide bond (19). *In vitro*, FXII can be activated by PK, plasmin, or on negatively charged surfaces, while *in vivo* activation is still under debate (20, 21). FXIIa is suggested to initiate the intrinsic coagulation, the contact, and complement systems (Figure 1). Thus, FXIIa leads to the cleavage of PK to generate active PK (contact system, Figure 2), triggers fibrin formation through the activation of factor XI (FXI; Figure 1), and activates the complement pathway. The serine protease C1 inhibitor (C1-INH) is the major inhibitor of FXII, and thereby controls its proteolytic activity. Besides C1-INH, antithrombin III and plasminogen activator inhibitor I also have FXII-inhibitory capacity. Despite its contribution to fibrin formation *in vitro*, FXII seems not to be essential for hemostasis *in vivo* (21, 22). However, under pathological conditions, FXII participates in thrombus formation and thromboembolic disorders, such as stroke (23).

In terms of neuroinflammation, we have been able to show that FXII deficiency leads to an attenuated disease severity in EAE, accompanied by reduced numbers of interleukin (IL)-17A-producing effector T helper cells (T_H_17). The role of FXII in EAE was mediated by its ability to shift the cytokine profile of dendritic cells (DC) necessary to induce differentiation of effector T cells (see also Table 1) (15). Pharmacologic inhibition of FXII by recombinant human albumin-tagged infestin-4 (24) resulted in decreased EAE severity as well (see Table 1). These findings suggest a potential novel link between FXII and the immune system in neuroinflammation. Strikingly, we also found significantly increased FXII plasma activity in individuals with relapsing–remitting MS and secondary progressive MS, as compared to healthy donors, thus, indicating a role for this factor in human MS pathogenesis (15).

**Table 1:** Studies of intrinsic and contact system factors: effects on neuroinflammatory processes in transgenic mice or using pharmacological substances.

Mouse line or treatment	Model (peptide)	Genetic background	Effects in models of autoimmune neurodegeneration	Reference
*B1R^−/−^*	Active EAE (MOG)	C57BL/6	Delayed EAE onset, reduced clinical deficits, reduced inflammation and demyelination, decreased expression of endothelial adhesion molecules, reduced migration of lymphocytes	(25)
			Earlier EAE onset, severe clinical disease course with enhanced inflammation, demyelination, glial activation, increased migration of CD4^+^ T cells, especially T_H_17 cells	(26)
			Delayed EAE onset, reduced clinical deficits, reduced inflammation and demyelination, decreased cytokine production of CD4^+^ T cells	(27)
			Delayed EAE onset, reduced clinical deficits, reduced glial activation, decreased release of proinflammatory mediators by astrocytes	(28)

Des-Arg^9^-BK (B1R activator)	Active EAE (MOG)	C57BL/6	No clinical effect	(27)

R838 (B1R agonist)	Active EAE (MOG)	C57BL/6	Aggravated disease course, enhanced inflammation, demyelination, axonal damage	(25)
			Milder clinical deficits	(26)
	Active RR-EAE (PLP)	SJL	Reduced clinical deficits	(26)

Des-Arg^9^-[Leu^8^]-BK (B1R inhibitor)	Active EAE (MOG)	C57BL/6	Delayed EAE onset, reduced clinical deficits, decreased release of proinflammatory mediators by astrocytes	(28)
			Delayed EAE onset, reduced clinical deficits, reduced inflammation and demyelination, decreased cytokine production of CD4^+^ T cells	(27)

R715 (B1R inhibitor)	Active EAE (MOG)	C57BL/6	Reduced clinical deficits, reduced inflammation, demyelination, axonal damage	(25)
			Accelerated disease onset	(26)

*B2R^−/−^*	Active EAE (MOG)	C57BL/6	No effect on clinical course, immune cells, cytokine production	(25, 26)
			Moderate reduced clinical deficits, reduced inflammation, reduced leukocyte adhesion, decreased chemokine (CCL2, CCL5) production	(29)

HOE-140 (B2R inhibitor)	Active EAE (MOG)	C57BL/6	Moderate or no effect on clinical disease course, immune cells, cytokine production	(25–28)
			Moderate reduced clinical deficits, reduced inflammation, reduced leukocyte adhesion, decreased chemokine (CCL2, CCL5) production	(29)

*F12^−/−^*	Active EAE (MOG)	C57BL/6	Delayed EAE onset, reduced clinical deficits, reduced inflammation and demyelination, reduced T_H_17 cells, decreased cytokine production (IL-6, IL-23) of DC	(15)

rHA-Infestin 4 (FXIIa inhibitor)	Active EAE (MOG)	C57BL/6	Delayed EAE onset, reduced clinical deficits, reduced inflammation and demyelination, reduced cytokine production (IL-6, IL-17A)	(15)
	Active EAE (PLP)	SJL	Ameliorated disease course	(15)

*F11^−/−^*	Active EAE (MOG)	C57BL/6	No effect on clinical symptoms, inflammation and demyelination	(15)

*C1q^−/−^*	Active EAE (MOG)	C57BL/6	No effect on clinical symptoms	(30)

*C3^−/−^*	Active EAE (MOG)	129SVJ/C57BL/6	Reduced clinical deficits, decreased inflammation and demyelination	(31)
			No clinical effect, but higher mortality, tendency to enhanced inflammation and demyelination	(32)

*C3^−/−^*	Active + AT-EAE (MOG)	C57BL/6	Reduced clinical deficits, reduced infiltration of T cells	(33, 34)

*C5^−/−^*	Active EAE (guinea pig myelin)	B10.D2/oSnJ	Moderate reduction of clinical deficits, narrow zones of inflammation and demyelination, gliosis, reduced remyelination, enhanced apoptosis of oligodendrocytes, axonal damage	(35, 36)

*C5a/GFAP*	Active EAE (MOG)	C57BL/6	No clinical effect	(37)

*Cd87^−/−^*	Active EAE (MOG)	C57BL/6	Aggravated disease course, enhanced inflammation, axonal damage, reduced T-cell proliferation and cytokine production	(38)
			Delayed disease onset, reduced clinical deficits, reduced inflammation, enhanced demyelination and axonal damage	(39)
	Active EAE (MOG) in BM-chimera		Reduced clinical deficits with *Cd87^−/−^* BM, partial protection of *Cd87^−/−^* hosts	(15)

*AT, adoptive transfer; B1R, bradykinin receptor 1; B2R, bradykinin receptor 2; BK, bradykinin; BM, bone marrow; CCL, chemokine (C–C) motif ligand; DC, dendritic cells; EAE; experimental autoimmune encephalomyelitis; FXIIa, activated factor XII; IL, interleukin; MOG, myelin oligodendrocyte glycoprotein; PLP, proteolipid protein; rHA, recombinant human albumin; RR, relapsing–remitting; T_H_17, IL-17A-producing effector T helper cells*.

As aforementioned, FXIIa leads to the cleavage of FXI (Figure 1). However, studies from our laboratory indicate that the latter factor has no significant role in EAE (see also Table 1), suggesting that not the entire intrinsic coagulation system is involved, but rather that the effect of FXII in neuroinflammation is dependent on other pathways triggered by FXII (15).

In particular, besides hemostasis, FXII leads to the activation of the contact system and hereby to the release of BK (Figure 2). Reports on the function of BK in MS and EAE remain contradictory. While three reports described a protective role of genetic or pharmacological inhibition of one distinct bradykinin receptor (bradykinin receptor 1, B1R), another study demonstrated enhanced inflammation by B1R blockade (see also Table 1) (25–28). For MS patients, B1R has been shown to have a detrimental effect, as it is upregulated on T-lymphocytes from patients with either secondary progressive MS or relapsing–remitting MS during active relapse (17). Levels of B1R expression on mononuclear cells correlate positively with the Expanded Disability Status Scale, with occurrence of clinical relapses and lesion volumes on T2-weighted images, but not with gadolinium-enhancing lesions (40). Furthermore, a potential role for B1R has been described in the regulation of blood–brain barrier permeability and chemokine production (18), indicating this factor’s involvement in neuroinflammation.

FXII has the capacity to activate the classic complement pathway by direct cleavage of C1q (20). However, it has been shown that C1q has no influence on neuroinflammation, at least in terms of clinical symptoms (30). It is known that PK can activate the complement components C3 and C5 (20). However, reports on these members of the complement system in the context of neuroinflammation remain elusive. While three reports showed a significant role of C3, as C3-deficient animals had an attenuated EAE disease course and reduced T-cell infiltration (31, 33, 34), another study showed no clinical differences, but a tendency to enhanced inflammation and demyelination (32). For C5, a dual role in EAE has been suggested: One study revealed that C5 leads to reduced inflammation and tissue repair in acute lesions, while this factor seemed to be responsible for increased axonal damage and enhanced gliosis in chronic lesions (35). Furthermore, it has been shown that C5 can limit oligodendrocyte apoptosis in EAE, thus promoting remyelination (36). Use of transgenic mice that express C5 under the astrocytic-specific glial fibrillary acidic protein promoter revealed no significant contribution to disease development of this component in the CNS (37), so that the role of complement in EAE remains contradictory.

**Figure 3:**
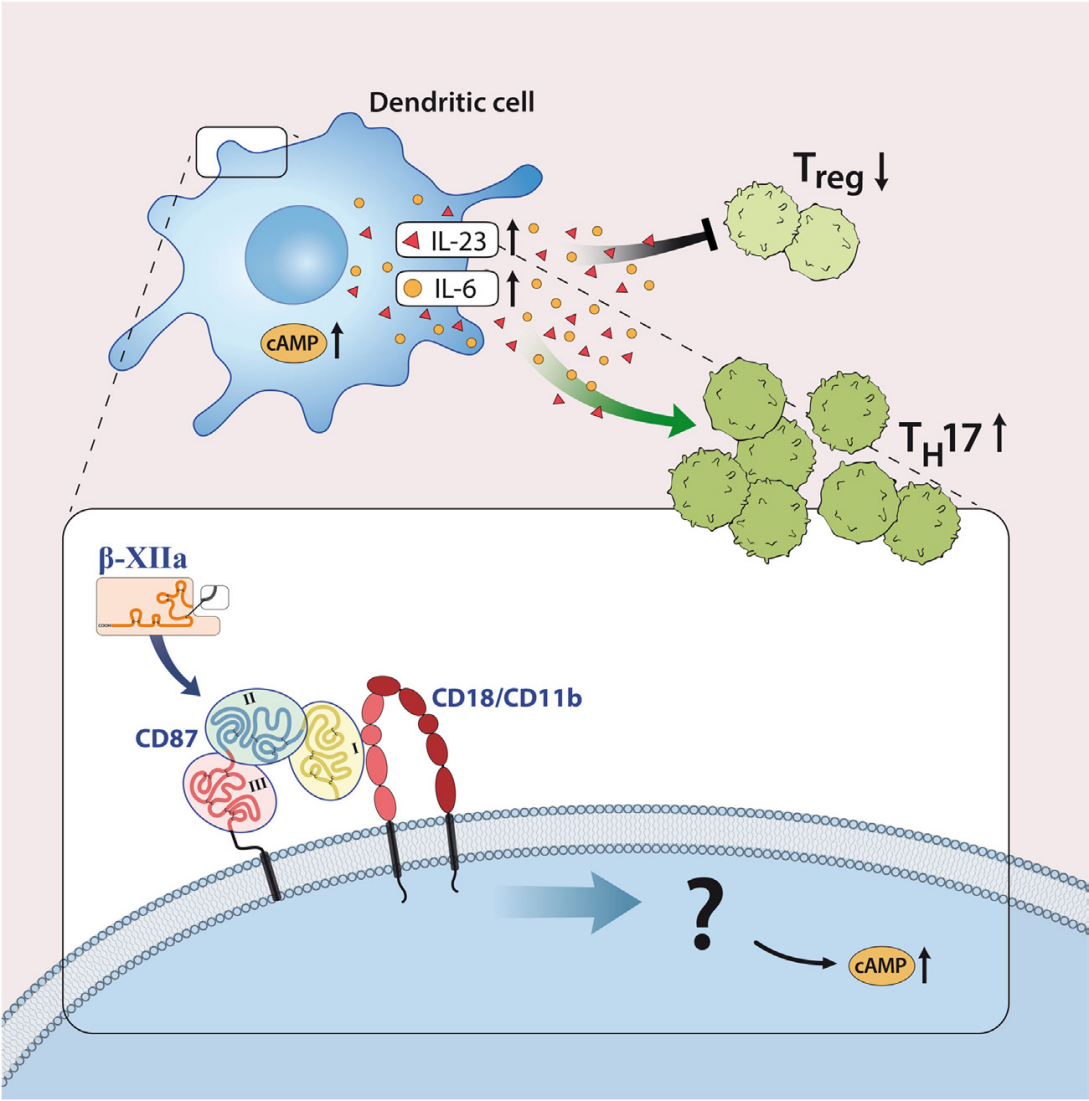
Factor XII (FXII) as a mediator of inflammatory disease. FXII acts on dendritic cells through the urokinase-type plasminogen-activator receptor (CD87) to enhance the release of interleukin (IL)-6 and -23. This cytokine shift leads to increased amounts of IL-17A-producing CD4^+^ effector T helper cells (T_H_17). Abbreviations: AC, adenylate cyclase; CD11b/CD18, leukocyte integrin adhesion molecule.

Although most investigations focus on FXII as a serine protease, FXII can interact with cells independently of its enzymatic activity. In line with this, FXII can bind to urokinase plasminogen activator receptor (uPAR, CD87; Figures 1 and 3) (20). Studies from our laboratory have demonstrated high levels of CD87 on DC. In this context, we could show that FXII exerts its immunoregulatory effects directly *via* CD87 and by regulating cyclic adenosine monophosphate (cAMP) and thereby cytokine levels (e.g., IL-6, IL-23) in DC. In contrast, we could rule out the involvement of alternative FXII-triggered pathways, such as the intrinsic coagulation, the contact and complement systems, for EAE pathogenesis.

However, reports on the relevance of CD87 inhibition, *per se*, in EAE remain contradictory: while two reports indicated a protective role in terms of clinical score and inflammation, when CD87 was missing (15, 39), and another showed enhanced inflammation (38).

In conclusion, the data so far indicate a significant role of FXII and downstream factors and pathways in neuroinflammation. However, further studies are needed to clarify remaining contradictions.

### Fibrinogen and Neuroinflammation

**Figure 4:**
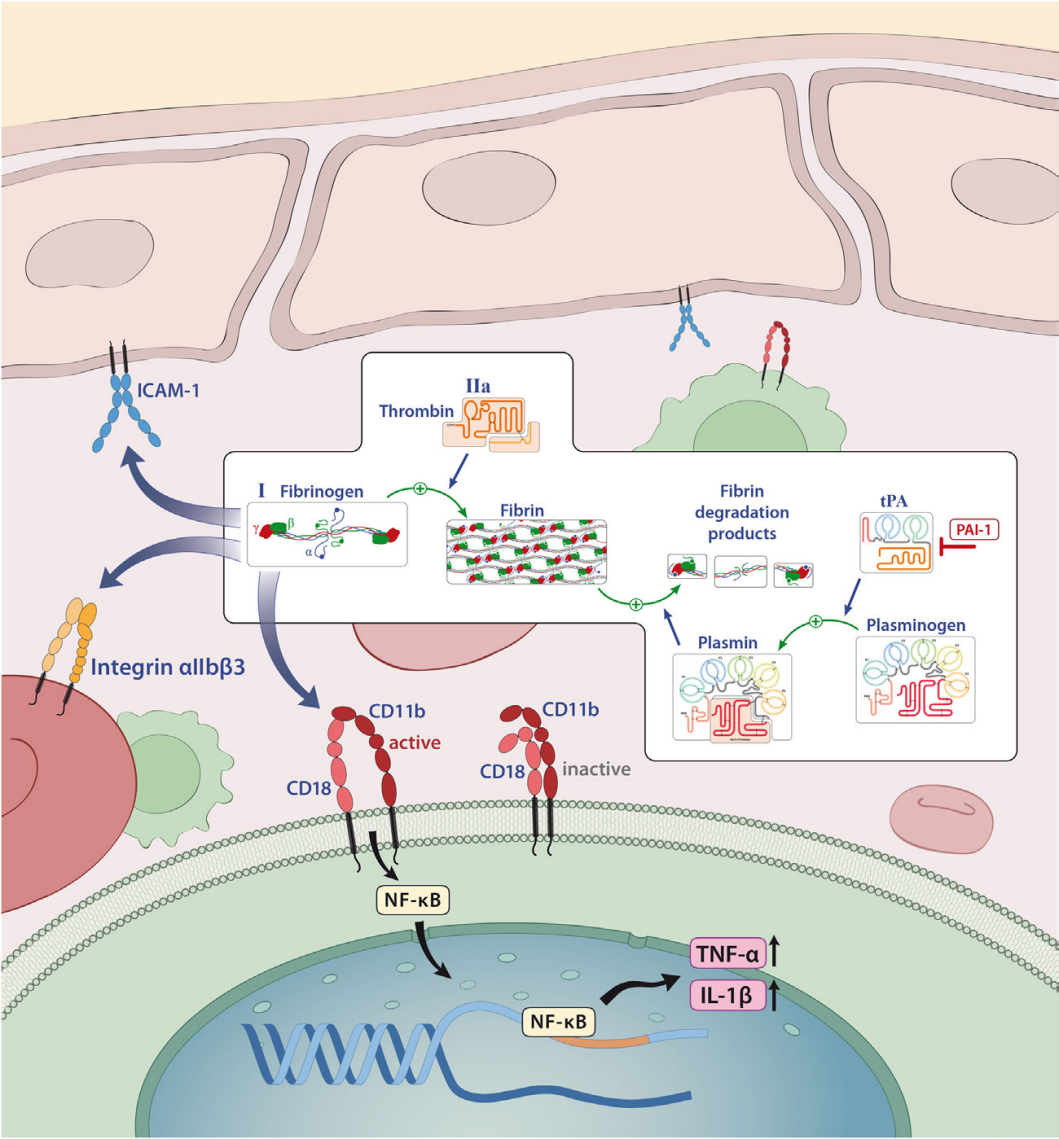
Fibrinogen as a mediator of inflammation. Fibrinogen acts on different cells through integrin and non-integrin receptors to induce specific inflammatory effects. Abbreviations: CD11b/CD18, leukocyte integrin adhesion molecule; ICAM-1, intercellular adhesion molecule-1; IL-1β, interleukin-1β; NF-κB, nuclear factor κB; tPA, tissue plasminogen activator; PAI-1, plasminogen-activator inhibitor 1; TNF-α, tumor necrosis factor α.

Fibrinogen (Factor I) is a 340-kDa glycoprotein that is synthesized in the liver (41). It is activated to fibrin by thrombin, exposing several polymerization sites that are crosslinked to an insoluble fibrin clot under the involvement of activated factor XIII (41, 42). Although activation of the coagulation system and thereby fibrin formation is essential for stopping lethal hemorrhage, the deposition of fibrin is carefully regulated to avoid thrombotic incidents (43). This is achieved by the fibrinolytic system in which plasmin especially counterbalances the procoagulatory signals, leads to clot dissolution, and results in the generation of soluble fibrin fragments, such as fragments D and E, and d-dimers (44). Plasmin generation is regulated by two proteases, tissue plasminogen activator (tPA) and uPA (45), which are controlled by plasminogen activator inhibitor-1 (PAI-1; Figures 1 and 4) (46).

Under physiological conditions, the plasma concentration of fibrinogen is between 2 and 4 g/l; however, it is known that this concentration can rapidly increase under pathological conditions (acute phase reactions), such as injury, infection, or inflammation (47, 48). Similarly, elevated levels of fibrin degradation products, such as d-dimer, are used in clinical practice as indicators of inflammation and risk predictors of thrombotic events (49). In the majority of cases, the proinflammatory function of fibrin/fibrinogen is mediated by its ability to bind to different immune cells for instance to the CD11b/CD18 integrin receptor (also termed Mac-1) on macrophages, monocytes, or microglia that induces the release of reactive oxygen species and is required for axonal damage in EAE (8, 47, 50). In this context, it has been shown that binding of fibrin/fibrinogen to the CD11b/CD18 integrin receptor results in activation of proinflammatory cascades, such as nuclear factor κB, which leads to the release of inflammatory cytokines, like tumor necrosis factor (TNF)-α or IL-1β (51, 52) and can thereby influence diseases such as RA (53) or colitis-associated cancer (Figure 4) (54). In addition, fibrinogen-dependent effects of platelets may also contribute to EAE disease pathogenesis (55).

A detrimental role of fibrin/fibrinogen has also been suggested for neuroinflammation, as fibrin deposition in the CNS correlates with microglial activation in active MS lesions (56, 57). In line with this finding, it has been shown that fibrinogen can directly activate microglia, enhance their phagocytic ability, induce peripheral macrophage recruitment and local CNS activation of myelin antigen-specific T_H_1 cells (58, 59). Moreover, genetic deletion of fibrinogen resulted in reduced inflammation and demyelination using a TNF transgenic model of MS (mice that lack the TNF receptor, develop spontaneous clinical symptoms of paralysis, and die by 5 weeks of age; *TgK21fib^−/−^*; see also Table 2) (60). Furthermore, inhibition of fibrinogen binding to CD11b/CD18 by genetic mutation of the CD11b/CD18-binding motif (*Fib*γ*^390–396A^*) (61) or a peptide (γ^377–395^) results in reduced microglial activation and an attenuated disease course in EAE (see also Table 2) (58).

**Table 2:** Studies of coagulation system factors: effects on neuroinflammatory processes using transgenic mice or pharmacological substances.

Mouse line (genetic background)	Model (peptide)	Genetic background or species	Neuroinflammatory effect	Reference
γ^377–395^	Active RR-EAE (PLP)	SJL	Reduced clinical symptoms, decreased inflammation, reduced microglial activation	(58)

Ancrod	Active RR-EAE (PLP)	SJL	Reduced clinical symptoms, decreased microglial activation and demyelination, no impact on immune cell proliferation	(58)
	TNF-transgenic model (no peptide, spontaneous)	Tg6072 TNF transgenic mice	Reduced demyelination, downregulation of MHC-I	(60)
	Active EAE	Rat	Reduced clinical symptoms, decreased inflammation and fibrin deposition	(62)

Batroxobin	Active EAE (MOG)	C57BL/6	Reduced clinical symptoms, decreased inflammation, demyelination, no effect on axonal damage	(63)
	AT-EAE (GP-MBP)	Rat	Attenuated disease course, reduced fibrin depositions, no effect on inflammation and immune cell proliferation	(64)

ε-Aminocaproic acid	Active EAE	Rat	Attenuated disease course	(65)

*Fib*γ*^390–396A^*	Active EAE (MOG)	C57BL/6	Reduced clinical symptoms, decreased inflammation, demyelination, reduced microglial activation	(58)

Hirudin	Active EAE (PLP)	SJL/J	Improvement of clinical deficits, reduced inflammation, decreased immune cell proliferation and cytokine production	(13)

*PAI-1^−/−^*	Active CREAE (spinal cord homogenate)	ABH	Reduced incidence, moderately delayed onset, reduced inflammation, no effect on demyelination and axonal damage	(39)

PAI-1-dp (uPA activator)	Active EAE (MOG)	C57BL/6	Attenuated disease course, reduced Tcell proliferation and cytokine production	(38)

*TgK21fib^−/−^*	TNF-transgenic model (no peptide, spontaneous)		Increased lifespan, delayed onset of clinical symptoms, reduced inflammation and demyelination	(60)

*tPA^−/−^*	Active EAE (MOG)	C57BL/6	Aggravated disease course, enhanced demyelination, axonal damage, fibrin deposition	(39)
			Delayed disease onset, aggravated disease course, delayed, but prolonged demyelination and axonal damage, reduced microglial activation	(66)

*uPA^−/−^*	Active EAE (MOG)	C57BL/6	Aggravated disease course, enhanced inflammation and microglial activation	(38)

*AT, adoptive transfer; CREAE, chronic relapsing experimental allergic encephalomyelitis; EAE, experimental autoimmune encephalomyelitis; MHC-I, major histocompatibility complex class I; MOG, myelin oligodendrocyte glycoprotein; PAI-I, plasminogen activator inhibitor I; PAI-I-dp, plasminogen activator inhibitor I-deprived; PLP, proteolipid protein; TNF, tumor necrosis factor; tPA, tissue plasminogen activator; uPA, urokinase plasminogen activator; RR, relapsing–remitting*.

Interestingly, none of these inhibitory approaches interferes with the clotting function of fibrinogen (53, 58). Moreover, staphylococcal-derived extracellular adherence protein, which, among others, interferes with the interaction between CD11b/CD18 and fibrinogen, also suppressed murine EAE disease severity (67), while pharmacological treatment strategies with snake venom-derived defibrinogenating agents, such as ancrod or batroxobin, suppress clinical symptoms in different animal models of MS (see also Table 2) (60, 62–64, 68).

Enhanced fibrin deposition is usually counterbalanced by plasmin that is generated by tPA and uPA. Interestingly, uPA as well as PAI-1 are significantly increased in acute MS lesions, while tPA levels are unchanged (69, 70). Results concerning tPA activity remain contradictory; while one report indicates a reduction in tPA activity in normal-appearing white and gray matter and lesions of individuals with MS (70), others describe a significant increase of activity in lesions and the cerebrospinal fluid of MS patients during the acute, but not the chronic disease phase (71).

When EAE was induced in *uPA^−/−^* mice, these animals displayed an aggravated disease course. This finding was accompanied by enhanced microglial activation (see also Table 2) (38). In line with these results, treatment with a PA inhibitor-derived peptide (PAI-1-dp) that increases plasminogen activation ability of uPA, suppressed the development of EAE symptoms (see also Table 2) (38). In contrast, another publication using ε-aminocaproic acid, an inhibitor of plasminogen and trypsinogen activator, reported a suppression of EAE severity (see also Table 2) (65).

Results of EAE experiments in tPA-deficient animals remain contradictory: while two publications described increased severity and a delayed recovery with enhanced demyelination and axonal damage after genetic depletion of tPA, disease onset was reported to be either earlier or delayed in the literature (see also Table 2) (39, 66). Due to the significant upregulation of PAI-1 in MS patients, EAE induced in PAI-1-deficient animals was shown to have moderate clinical protection with reduced perivascular cuffs, but no difference in terms of demyelination or axonal damage was observed (39).

Nonetheless, data so far indicate a significant role of local fibrin deposits in neuroinflammation and indicate a promising anti-inflammatory therapeutic potential of targeting this pathway.

### Thrombin and Neuroinflammation

Prothrombin (factor II) is a soluble 72-kDa protein that is produced by the liver. It is activated to thrombin (factor IIa) *via* enzymatic cleavage of two sites by activated FX (FXa). Activated thrombin leads to cleavage of fibrinogen into fibrin monomers that, upon polymerization, form a fibrin clot. Therefore, activation of prothrombin is crucial in physiological and pathophysiological coagulation. For instance, various rare disorders, such as congenital hypoprothrombinemia (a blood disease in which deficiency of prothrombin results in impaired blood clotting) and acquired hypoprothrombinemia (e.g., in autoimmune diseases with lupus anticoagulant) have been described (72, 73).

Beyond its key role in coagulation, thrombin can mediate further effects, e.g., thrombin is a potent vasoconstrictor and is implicated in vasospasms following subarachnoid hemorrhage (74).

In terms of neuroinflammation, thrombin activity was found to be significantly increased in the spinal cord of mice with EAE (14). Thrombin activity precedes the onset of neurological signs and correlates with the amount of fibrin deposition, microglial activation, demyelination, axonal damage, and clinical severity. Interestingly, inhibition of thrombin activity by hirudin leads to a significant improvement of disease severity (13) (see also Table 2). This is accompanied by decreased immune cell proliferation and cytokine secretion, as well as a reduction in the number of inflammatory lesions (13). Furthermore, it has been shown that levels of thrombin inhibitors are significantly increased during EAE. For instance, antithrombin III (as well as protease nexin 1) were detected at higher levels in CNS homogenates during EAE compared with controls (75). Additionally, it was recently shown that prothrombin levels are elevated in plasma of patients suffering from relapsing-remitting MS or secondary progressive MS indicating a prominent role of this coagulation factor in neuroinflammation (16).

### Coagulation Factors in Non-Neurological Inflammatory Diseases

An increasing body of evidence also supports a decisive role of coagulation factors in regulating inflammatory responses in non-neurological inflammatory diseases. For instance, a substantial contribution of different coagulation factors has been suggested in RA or inflammatory joint disease as fibrin depositions can be found in the joints of patients with RA (76). Moreover, the degradation products of fibrin, such as d-dimer, are used as common biomarkers for disease activity (77, 78). *In vitro*, it was shown that fibrinogen can enhance IL-8 secretion and intercellular adhesion molecule 1 expression from human synovial fibroblasts, leading to enhanced lymphocyte adhesiveness (79). A further direct proinflammatory role of fibrin/fibrinogen was suggested in RA pathogenesis as its genetic depletion in mice leads to an improvement in the clinical symptoms in animal models of RA and results in decreased synovial inflammation (see Table 3) (53). Interestingly, it was shown that the interaction of fibrinogen with immune cells *via* CD11b/CD18 is the relevant partner for this effect. Furthermore, pharmacological inhibition of thrombin *via* hirudin resulted in a significant reduction in synovial inflammation and disease severity in two different animal models of RA (see Table 3) (80, 81). In this context, it could be shown that the plasmin activity is decreased, while PAI-1 levels are increased in both blood and inflamed joints of mice with collagen-induced arthritis (CIA) (82).

**Table 3:** Studies of coagulation system factors: effects on inflammatory processes using transgenic mice or pharmacological substances.

Mouse line or treatment	Model (peptide)	Genetic background or species	Effect in arthritis and colitis models	Reference
*B1R/B2R^−/−^*	CAIA (anti-CII antibodies)	C57BL/6	Decreased clinical symptoms, reduced inflammation and cytokine levels	(83)

C11C1 (HMWK antibody)	Reactive arthritis (PG-PS)	Lewis rats	Reduced joint diameter, local inflammation	(84)
	Spontaneous arthritis	HLA-B27 transgenic rats	Reduced joint destruction, inflammation	(85)

*Fib^−/−^*	CIA (type II collagen)	DBA/1J	Reduced incidence, severity, joint destruction, synovial inflammation	(53)

*Fib^−/−^*	Colitis-associated cancer (DSS)	C57BL/6	Reduced inflammation-driven adenoma formation	(54)

*Fib*^Δ^*^5^*	CIA (type II collagen)	DBA/1J	No effect on incidence and severity	(53)

*Fib*γ*^390–396A^*	CIA (type II collagen)	DBA/1J	Reduced incidence, severity, joint destruction, synovial inflammation	(53)

*Fib*γ*^390–396A^*	Colitis (DSS)	C57BL/6	Diminished inflammatory disease, reduced ulceration, cytokine levels and neutrophil infiltration	(54)

*Fib*γ*^390–396A^*	Colitis-associated cancer (DSS)	C57BL/6	Reduced inflammation-driven adenoma formation	(54)

Heparin	CIA (type II collagen)	DBA/1	No effect on clinical score	(82)

Hirudin	AIA (mBSA)	C57BL/6	Reduced knee joint inflammation, fibrin deposition	(80)

	CIA (type II collagen)	DBA/1J	Decreased disease incidence, severity, reduced loss of articular cartilage, inflammation, fibrin deposition and PAR-1 expression	(81)

*HMWK^−/−^*	Reactive arthritis (PG-PS)	Rats	No clinical signs of arthritis, absence of inflammation	(86)

MEN16132 (B2R antagonist)	Inflammatory arthritis (carrageenan)	Rats	Reduced knee diameter and myeloperoxidase activity	(87)

P8720 (PK inhibitor)	Reactive arthritis (PG-PS)	Lewis rats	Decreased joint swelling, reduced inflammation	(88)

PKSI-527 (PK inhibitor)	CIA (type II collagen)	DBA/1	Reduced severity of arthritis, reduced PK and HMWK plasma levels	(89)

*Plg^−/−^*	AIA (mBSA)	C57BL/6	Enhanced inflammation, bone erosion, synovial thickness, fibrin deposition	(90)

*Plg^−/−^*	CIA (type II collagen)	C57BL/6xDBA/1	No clinical symptoms of disease, no signs of inflammation	(91)
	CAIA (anti-CII antibodies)		No clinical symptoms of disease, no signs of inflammation	(91)
	AIA (mBSA)		Enhanced synovial thickness	(92)
	CIA (type II collagen)		Reduced synovial thickness	(92)

*Tg197Fib^−/−^*	TNF-transgenic model, spontaneous polyarthritis		No effect on incidence, severity, inflammation, joint destruction	(53)

uPA	CIA (type II collagen)	DBA/1	No effect on clinical score, reduced fibrin deposits in joints, decreased plasma d-dimer level	(82)

*uPA^−/−^*	AIA (mBSA)	Ola129xC57BL/6	Enhanced joint inflammation, bone erosion, synovial thickness, fibrin deposition	(90)

*uPA^−/−^*	Monoarticular arthritis (mBSA/IL-1)	C57BL/6	Enhanced arthritis, fibrin deposition, increased numbers of macrophages	(93)

	CIA (type II collagen)		Reduced clinical symptoms, decreased inflammation, cartilage destruction, bone erosion, reduced cytokine production	(94)

	CAIA (M2139, CIIC1 antibodies)		No clinical signs of disease	(95)

	AIA (mBSA)		Enhanced proteoglycan loss, inflammation, bone erosion	(92, 96)

*uPA^−/−^*	CIA (type II collagen)	C57BL/6xDBA/1	Reduced clinical symptoms, no histological changes	(91)

	K/BxN serum transfer arthritis (K/BxN serum)		No clinical signs of disease, reduced inflammation, bone erosion, fibrin deposition	(95, 96)

tPA	CIA (type II collagen)	DBA/1	No effect on clinical score, reduced fibrin deposits in joints	(82)

*tPA^−/−^*	Monoarticular arthritis (mBSA/IL-1)	C57BL/6	Enhanced arthritis, fibrin deposition, increased numbers of macrophages	(93)

	CIA (type II collagen)		Aggravated clinical symptoms, enhanced inflammation, cartilage destruction, bone erosion, fibrin deposition	(94)

*AIA, antigen-induced arthritis; B1R, bradykinin receptor I; B2R, bradykinin receptor 2; CAIA, type II collagen mAb-induced arthritis; CIA, collagen-induced arthritis; CII, collagen type II; DSS, dextran sulfate sodium; HLA, human leukocyte antigen; HMWK, high molecular weight kininogen; mBSA, methylated bovine serum albumin; PAR-1, protease-activated receptor; PG-PS, peptidoglycan-polysaccharide; PK, plasma kallikrein; TNF, tumor necrosis factor; tPA, tissue plasminogen activator; uPA, urokinase plasminogen activator*.

While treatment with uPA and tPA improves plasmin activity and removes fibrinogen depositions in joints, disease severity remains unchanged, challenging the pathophysiological role of fibrinogen in this context (82). Nonetheless, a significant contribution of uPA could be seen in other studies, but this remains contradictory for different arthritis models: in monoarticular models, uPA-deficient mice had an aggravated disease course (90, 93). In contrast, other studies using polyarticular animal models demonstrated resistance to or suppression of disease and reduced inflammation in animals lacking uPA, indicating a distinct role of uPA in different types of arthritis (91, 92, 94–96). The same result was observed in plasminogen-deficient animals (90–92). In contrast to uPA, studies using tPA-deficient animals have so far indicated an aggravated disease course with enhanced inflammation (93, 94).

A substantial role for the contact system in arthritis has been discussed. For instance, FXIIa levels were significantly increased in RA patients compared with healthy controls (97). Furthermore, pharmacological blockade of PK by different inhibitors revealed reduced disease severity and inflammation in different models of arthritis (88, 89). In line with these findings, genetic or pharmacological inhibition of HMWK leads to an attenuation of PK–kinin system activation, local and systemic inflammation, indicating a therapeutic potential in RA (84–86). Moreover, arthritis severity is significantly attenuated in mice lacking B1R and B2R (83) or by treatment with a B2R antagonist (87, 98).

In addition to RA, potential drugability of the coagulation system and its factors is under consideration for the treatment of inflammatory bowel disease. Interestingly, it was shown that patients with Crohn’s disease have significantly higher levels of C1-inhibitor and intestinal tissue kallikrein, while plasma levels of prekallikrein, FXI, and HMWK are unaltered (99). Furthermore, inflammatory bowel disease in humans is associated with higher plasma levels of fibrinogen, prothrombin, factor V, factor VIII, plasminogen, and platelets (100). In line with these findings, animal models of colitis have demonstrated reduced inflammation in animals with a genetic disruption to the binding of fibrinogen to the CD11b/CD18 integrin receptor (see Table 3) (54). Since a link between chronic inflammation and tumor development, e.g., colitis and colorectal cancer, could be established (101), it is interesting that both fibrinogen-deficient mice and mice with a genetic disruption of the interaction between fibrinogen and the CD11b/CD18 integrin receptor develop significantly fewer adenomas (54).

Collectively, these results demonstrate a clear role of the coagulation system, not only in neuroinflammation, but also in other autoimmune and inflammatory disorders.

## Conclusion

In this review, we have discussed the links between coagulation and inflammation, focusing on the role of different coagulation factors in neuroinflammatory disorders. Overall, it becomes increasingly clear that the deposition of different coagulation factors in the CNS tissue may trigger exacerbation of inflammation, thereby limiting regenerative mechanisms. A prominent role was especially described for fibrinogen, thrombin, and factor XII. As novel molecular and cellular binding partners are identified, the role of coagulation factors is evolving from hemostasis regulators to multi-faceted signal molecules, which control the balance between immune defense mechanisms and extensive inflammation.

Interestingly, the binding of coagulation factors to their cellular targets requires distinct non-overlapping epitopes and is usually independent of their protease function. Taking advantage of this knowledge, targeted inhibition of coagulation factors that facilitate disease pathogenesis without affecting their protease activity represents an ideal strategy for pharmacological intervention in different neuroinflammatory disorders without unwarranted side-effects like bleeding. Therefore, future studies are needed to elucidate the exact contribution of blood proteins to autoimmune neurodegeneration.

## Author Contributions

KG drafted the manuscript. SE, HW, TC, CK, and SM extensively revised the manuscript. KG and TC funded the study. All authors provided substantial input throughout the process.

## Conflict of Interest Statement

The authors declare that the research was conducted in the absence of any commercial or financial relationships that could be construed as a potential conflict of interest.

## References

[1] Macfarlane RG An enzyme cascade in the blood clotting mechanism, and its function as a biochemical amplifier. Nature (1964) 202:498–9. 10.1038/202498a0 14167839

[2] Schoenmakers SH Reitsma PH Spek CA. Blood coagulation factors as inflammatory mediators. Blood Cells Mol Dis (2005) 34(1):30–7. 10.1016/j.bcmd.2004.09.001 15607697

[3] McMichael M. New models of hemostasis. Top Companion Anim Med (2012) 27(2):40–5. 10.1053/j.tcam.2012.07.005 23031454

[4] Gailani D Broze GJ Jr. Factor XI activation in a revised model of blood coagulation. Science (1991) 253(5022):909–12. 10.1126/science.1652157 1652157

[5] Lippi G Favaloro EJ Franchini M Guidi GC. Milestones and perspectives in coagulation and hemostasis. Semin Thromb Hemost (2009) 35(1):9–22. 10.1055/s-0029-1214144 19308889

[6] Strukova SM. Thrombin as a regulator of inflammation and reparative processes in tissues. Biochemistry (Mosc) (2001) 66(1):8–18. 10.1023/A:1002869310180 11240387

[7] Mackman N. The many faces of tissue factor. J Thromb Haemost (2009) 7(Suppl 1):136–9. 10.1111/j.1538-7836.2009.03368.x 19630786 PMC2834482

[8] Davalos D Akassoglou K. Fibrinogen as a key regulator of inflammation in disease. Semin Immunopathol (2012) 34(1):43–62. 10.1007/s00281-011-0290-8 22037947

[9] Chavakis T Boeckel N Santoso S Voss R Isordia-Salas I Pixley RA Inhibition of platelet adhesion and aggregation by a defined region (Gly-486-Lys-502) of high molecular weight kininogen. J Biol Chem (2002) 277(26):23157–64. 10.1074/jbc.M202529200 11970955

[10] Chavakis T Pixley RA Isordia-Salas I Colman RW Preissner KT A novel antithrombotic role for high molecular weight kininogen as inhibitor of plasminogen activator inhibitor-1 function. J Biol Chem (2002) 277(36):32677–82. 10.1074/jbc.M204010200 12082110

[11] Chavakis T Kanse SM Pixley RA May AE Isordia-Salas I Colman RW Regulation of leukocyte recruitment by polypeptides derived from high molecular weight kininogen. FASEB J (2001) 15(13):2365–76. 10.1096/fj.01-0201com 11689462

[12] Leeb-Lundberg LM Marceau F Muller-Esterl W Pettibone DJ Zuraw BL. International union of pharmacology. XLV. Classification of the kinin receptor family: from molecular mechanisms to pathophysiological consequences. Pharmacol Rev (2005) 57(1):27–77. 10.1124/pr.57.1.2 15734727

[13] Han MH Hwang SI Roy DB Lundgren DH Price JV Ousman SS Proteomic analysis of active multiple sclerosis lesions reveals therapeutic targets. Nature (2008) 451(7182):1076–81. 10.1038/nature06559 18278032

[14] Davalos D Baeten KM Whitney MA Mullins ES Friedman B Olson ES Early detection of thrombin activity in neuroinflammatory disease. Ann Neurol (2014) 75(2):303–8. 10.1002/ana.24078 24740641 PMC4049631

[15] Gobel K Pankratz S Asaridou CM Herrmann AM Bittner S Merker M Blood coagulation factor XII drives adaptive immunity during neuroinflammation via CD87-mediated modulation of dendritic cells. Nat Commun (2016) 7:11626 10.1038/ncomms11626 27188843 PMC4873982

[16] Gobel K Kraft P Pankratz S Gross CC Korsukewitz C Kwiecien R Prothrombin and factor X are elevated in multiple sclerosis patients. Ann Neurol (2016) 80(6):946–51. 10.1002/ana.24807 27774643

[17] Prat A Weinrib L Becher B Poirier J Duquette P Couture R Bradykinin B1 receptor expression and function on T lymphocytes in active multiple sclerosis. Neurology (1999) 53(9):2087–92. 10.1212/WNL.53.9.2087 10599786

[18] Prat A Biernacki K Pouly S Nalbantoglu J Couture R Antel JP. Kinin B1 receptor expression and function on human brain endothelial cells. J Neuropathol Exp Neurol (2000) 59(10):896–906. 10.1093/jnen/59.10.896 11079780

[19] Stavrou E Schmaier AH Factor XII: what does it contribute to our understanding of the physiology and pathophysiology of hemostasis & thrombosis. Thromb Res (2010) 125(3):210–5. 10.1016/j.thromres.2009.11.028 20022081 PMC2851158

[20] Schmaier AH. The elusive physiologic role of Factor XII. J Clin Invest (2008) 118(9):3006–9. 10.1172/JCI36617 18725991 PMC2518076

[21] Renne T Schmaier AH Nickel KF Blomback M Maas C. In vivo roles of factor XII. Blood (2012) 120(22):4296–303. 10.1182/blood-2012-07-292094 22993391 PMC3507141

[22] Spronk HM Dielis AW Panova-Noeva M van Oerle R Govers-Riemslag JW Hamulyak K Monitoring thrombin generation: is addition of corn trypsin inhibitor needed? Thromb Haemost (2009) 101(6):1156–62. 10.1160/TH08-10-0670 19492161

[23] Kleinschnitz C Stoll G Bendszus M Schuh K Pauer HU Burfeind P Targeting coagulation factor XII provides protection from pathological thrombosis in cerebral ischemia without interfering with hemostasis. J Exp Med (2006) 203(3):513–8. 10.1084/jem.20052458 16533887 PMC2118228

[24] Hagedorn I Schmidbauer S Pleines I Kleinschnitz C Kronthaler U Stoll G Factor XIIa inhibitor recombinant human albumin Infestin-4 abolishes occlusive arterial thrombus formation without affecting bleeding. Circulation (2010) 121(13):1510–7. 10.1161/CIRCULATIONAHA.109.924761 20308613

[25] Gobel K Pankratz S Schneider-Hohendorf T Bittner S Schuhmann MK Langer HF Blockade of the kinin receptor B1 protects from autoimmune CNS disease by reducing leukocyte trafficking. J Autoimmun (2011) 36(2):106–14. 10.1016/j.jaut.2010.11.004 21216565

[26] Schulze-Topphoff U Prat A Prozorovski T Siffrin V Paterka M Herz J Activation of kinin receptor B1 limits encephalitogenic T lymphocyte recruitment to the central nervous system. Nat Med (2009) 15(7):788–93. 10.1038/nm.1980 19561616 PMC4903020

[27] Dutra RC Leite DF Bento AF Manjavachi MN Patricio ES Figueiredo CP The role of kinin receptors in preventing neuroinflammation and its clinical severity during experimental autoimmune encephalomyelitis in mice. PLoS One (2011) 6(11):e27875. 10.1371/journal.pone.0027875 22132157 PMC3222659

[28] Dutra RC Bento AF Leite DF Manjavachi MN Marcon R Bicca MA The role of kinin B1 and B2 receptors in the persistent pain induced by experimental autoimmune encephalomyelitis (EAE) in mice: evidence for the involvement of astrocytes. Neurobiol Dis (2013) 54:82–93. 10.1016/j.nbd.2013.02.007 23454198

[29] Dos Santos AC Roffe E Arantes RM Juliano L Pesquero JL Pesquero JB Kinin B2 receptor regulates chemokines CCL2 and CCL5 expression and modulates leukocyte recruitment and pathology in experimental autoimmune encephalomyelitis (EAE) in mice. J Neuroinflammation (2008) 5:49. 10.1186/1742-2094-5-49 18986535 PMC2596102

[30] Urich E Gutcher I Prinz M Becher B. Autoantibody-mediated demyelination depends on complement activation but not activatory Fc-receptors. Proc Natl Acad Sci U S A (2006) 103(49):18697–702. 10.1073/pnas.0607283103 17121989 PMC1693725

[31] Nataf S Carroll SL Wetsel RA Szalai AJ Barnum SR. Attenuation of experimental autoimmune demyelination in complement-deficient mice. J Immunol (2000) 165(10):5867–73. 10.4049/jimmunol.165.10.5867 11067947

[32] Calida DM Constantinescu C Purev E Zhang GX Ventura ES Lavi E Cutting edge: C3, a key component of complement activation, is not required for the development of myelin oligodendrocyte glycoprotein peptide-induced experimental autoimmune encephalomyelitis in mice. J Immunol (2001) 166(2):723–6. 10.4049/jimmunol.166.2.723 11145641

[33] Szalai AJ Hu X Adams JE Barnum SR. Complement in experimental autoimmune encephalomyelitis revisited: C3 is required for development of maximal disease. Mol Immunol (2007) 44(12):3132–6. 10.1016/j.molimm.2007.02.002 17353050 PMC1986644

[34] Smith SS Ludwig M Wohler JE Bullard DC Szalai AJ Barnum SR. Deletion of both ICAM-1 and C3 enhances severity of experimental autoimmune encephalomyelitis compared to C3-deficient mice. Neurosci Lett (2008) 442(2):158–60. 10.1016/j.neulet.2008.07.005 18634851 PMC2556246

[35] Weerth SH Rus H Shin ML Raine CS. Complement C5 in experimental autoimmune encephalomyelitis (EAE) facilitates remyelination and prevents gliosis. Am J Pathol (2003) 163(3):1069–80. 10.1016/S0002-9440(10)63466-9 12937147 PMC1868269

[36] Niculescu T Weerth S Niculescu F Cudrici C Rus V Raine CS Effects of complement C5 on apoptosis in experimental autoimmune encephalomyelitis. J Immunol (2004) 172(9):5702–6. 10.4049/jimmunol.172.9.5702 15100315

[37] Reiman R Campos Torres A Martin BK Ting JP Campbell IL Barnum SR Expression of C5a in the brain does not exacerbate experimental autoimmune encephalomyelitis. Neurosci Lett (2005) 390(3):134–8. 10.1016/j.neulet.2005.08.022 16154690

[38] Gur-Wahnon D Mizrachi T Maaravi-Pinto FY Lourbopoulos A Grigoriadis N Higazi AA The plasminogen activator system: involvement in central nervous system inflammation and a potential site for therapeutic intervention. J Neuroinflammation (2013) 10:124. 10.1186/1742-2094-10-124 24120085 PMC3852474

[39] East E Baker D Pryce G Lijnen HR Cuzner ML Gveric D. A role for the plasminogen activator system in inflammation and neurodegeneration in the central nervous system during experimental allergic encephalomyelitis. Am J Pathol (2005) 167(2):545–54. 10.1016/S0002-9440(10)62996-3 16049338 PMC1603566

[40] Prat A Biernacki K Saroli T Orav JE Guttmann CR Weiner HL Kinin B1 receptor expression on multiple sclerosis mononuclear cells: correlation with magnetic resonance imaging T2-weighted lesion volume and clinical disability. Arch Neurol (2005) 62(5):795–800. 10.1001/archneur.62.5.795 15883268

[41] Weisel JW. Fibrinogen and fibrin. Adv Protein Chem (2005) 70:247–99. 10.1016/S0065-3233(05)70008-5 15837518

[42] Fuss C Palmaz JC Sprague EA. Fibrinogen: structure, function, and surface interactions. J Vasc Interv Radiol (2001) 12(6):677–82. 10.1016/S1051-0443(07)61437-7 11389218

[43] Mosesson MW Dysfibrinogenemia and thrombosis. Semin Thromb Hemost (1999) 25(3):311–9. 10.1055/s-2007-994933 10443961

[44] Sidelmann JJ Gram J Jespersen J Kluft C. Fibrin clot formation and lysis: basic mechanisms. Semin Thromb Hemost (2000) 26(6):605–18. 10.1055/s-2000-13216 11140797

[45] Lijnen HR. Elements of the fibrinolytic system. Ann N Y Acad Sci (2001) 936:226–36. 10.1111/j.1749-6632.2001.tb03511.x 11460480

[46] East E Gveric D Baker D Pryce G Lijnen HR Cuzner ML. Chronic relapsing experimental allergic encephalomyelitis (CREAE) in plasminogen activator inhibitor-1 knockout mice: the effect of fibrinolysis during neuroinflammation. Neuropathol Appl Neurobiol (2008) 34(2):216–30. 10.1111/j.1365-2990.2007.00889.x 17983428

[47] Adams RA Passino M Sachs BD Nuriel T Akassoglou K. Fibrin mechanisms and functions in nervous system pathology. Mol Interv (2004) 4(3):163–76. 10.1124/mi.4.3.6 15210870

[48] Tennent GA Brennan SO Stangou AJ O’Grady J Hawkins PN Pepys MB Human plasma fibrinogen is synthesized in the liver. Blood (2007) 109(5):1971–4. 10.1182/blood-2006-08-040956 17082318

[49] Lowe GD Fibrinogen measurement to assess the risk of arterial thrombosis in individual patients: not yet. J Thromb Haemost (2005) 3(4):635–7. 10.1111/j.1538-7836.2005.01256.x 15842345

[50] Solovjov DA Pluskota E Plow EF. Distinct roles for the alpha and beta subunits in the functions of integrin alphaMbeta2. J Biol Chem (2005) 280(2):1336–45. 10.1074/jbc.M406968200 15485828

[51] Perez RL Roman J. Fibrin enhances the expression of IL-1 beta by human peripheral blood mononuclear cells. Implications in pulmonary inflammation. J Immunol (1995) 154(4):1879–87. 7836771

[52] Perez RL Ritzenthaler JD Roman J. Transcriptional regulation of the interleukin-1beta promoter via fibrinogen engagement of the CD18 integrin receptor. Am J Respir Cell Mol Biol (1999) 20(5):1059–66. 10.1165/ajrcmb.20.5.3281 10226077

[53] Flick MJ LaJeunesse CM Talmage KE Witte DP Palumbo JS Pinkerton MD Fibrin(ogen) exacerbates inflammatory joint disease through a mechanism linked to the integrin alphaMbeta2 binding motif. J Clin Invest (2007) 117(11):3224–35. 10.1172/JCI30134 17932565 PMC2000806

[54] Steinbrecher KA Horowitz NA Blevins EA Barney KA Shaw MA Harmel-Laws E Colitis-associated cancer is dependent on the interplay between the hemostatic and inflammatory systems and supported by integrin alpha(M)beta(2) engagement of fibrinogen. Cancer Res (2010) 70(7):2634–43. 10.1158/0008-5472.CAN-09-3465 20233870 PMC4288842

[55] Langer HF Choi EY Zhou H Schleicher R Chung KJ Tang Z Platelets contribute to the pathogenesis of experimental autoimmune encephalomyelitis. Circ Res (2012) 110(9):1202–10. 10.1161/CIRCRESAHA.111.256370 22456181 PMC3382058

[56] Wakefield AJ More LJ Difford J McLaughlin JE. Immunohistochemical study of vascular injury in acute multiple sclerosis. J Clin Pathol (1994) 47(2):129–33. 10.1136/jcp.47.2.129 8132826 PMC501826

[57] Gay FW Drye TJ Dick GW Esiri MM. The application of multifactorial cluster analysis in the staging of plaques in early multiple sclerosis. Identification and characterization of the primary demyelinating lesion. Brain (1997) 120(Pt 8):1461–83. 10.1093/brain/120.8.1461 9278635

[58] Adams RA Bauer J Flick MJ Sikorski SL Nuriel T Lassmann H The fibrin-derived gamma377-395 peptide inhibits microglia activation and suppresses relapsing paralysis in central nervous system autoimmune disease. J Exp Med (2007) 204(3):571–82. 10.1084/jem.20061931 17339406 PMC2137908

[59] Ryu JK Petersen MA Murray SG Baeten KM Meyer-Franke A Chan JP Blood coagulation protein fibrinogen promotes autoimmunity and demyelination via chemokine release and antigen presentation. Nat Commun (2015) 6:8164. 10.1038/ncomms9164 26353940 PMC4579523

[60] Akassoglou K Adams RA Bauer J Mercado P Tseveleki V Lassmann H Fibrin depletion decreases inflammation and delays the onset of demyelination in a tumor necrosis factor transgenic mouse model for multiple sclerosis. Proc Natl Acad Sci U S A (2004) 101(17):6698–703. 10.1073/pnas.0303859101 15096619 PMC404108

[61] Flick MJ Du X Witte DP Jirouskova M Soloviev DA Busuttil SJ Leukocyte engagement of fibrin(ogen) via the integrin receptor alphaMbeta2/Mac-1 is critical for host inflammatory response in vivo. J Clin Invest (2004) 113(11):1596–606. 10.1172/JCI20741 15173886 PMC419487

[62] Paterson PY Experimental allergic encephalomyelitis: role of fibrin deposition in immunopathogenesis of inflammation in rats. Fed Proc (1976) 35(13):2428–34. 61895

[63] Yang Y Tian SJ Wu L Huang DH Wu WP. Fibrinogen depleting agent batroxobin has a beneficial effect on experimental autoimmune encephalomyelitis. Cell Mol Neurobiol (2011) 31(3):437–48. 10.1007/s10571-010-9637-2 21165693 PMC11498529

[64] Inoue A Koh CS Shimada K Yanagisawa N Yoshimura K. Suppression of cell-transferred experimental autoimmune encephalomyelitis in defibrinated Lewis rats. J Neuroimmunol (1996) 71(1–2):131–7. 10.1016/S0165-5728(96)00150-6 8982112

[65] Sibley WA Kiernat J Laguna JF. The modification of experimental allergic encephalomyelitis with epsilon aminocaproic acid. Neurology (1978) 28(9 Pt 2):102–5. 10.1212/WNL.28.9_Part_2.102 568743

[66] Lu W Bhasin M Tsirka SE. Involvement of tissue plasminogen activator in onset and effector phases of experimental allergic encephalomyelitis. J Neurosci (2002) 22(24):10781–9. 10.1523/JNEUROSCI.22-24-10781.2002 12486171 PMC4002885

[67] Xie C Alcaide P Geisbrecht BV Schneider D Herrmann M Preissner KT Suppression of experimental autoimmune encephalomyelitis by extracellular adherence protein of *Staphylococcus aureus*. J Exp Med (2006) 203(4):985–94. 10.1084/jem.20051681 16585266 PMC2118278

[68] Paterson PY Experimental allergic encephalomyelitis-inducing activity of synthetic polyadenylic and polyuridylic homopolymers and complexes in guinea pigs. Cell Immunol (1976) 21(1):48–55. 10.1016/0008-8749(76)90326-9 55310

[69] Gveric D Hanemaaijer R Newcombe J van Lent NA Sier CF Cuzner ML. Plasminogen activators in multiple sclerosis lesions: implications for the inflammatory response and axonal damage. Brain (2001) 124(Pt 10):1978–88. 10.1093/brain/124.10.1978 11571216

[70] Gveric D Herrera B Petzold A Lawrence DA Cuzner ML. Impaired fibrinolysis in multiple sclerosis: a role for tissue plasminogen activator inhibitors. Brain (2003) 126(Pt 7):1590–8. 10.1093/brain/awg167 12805124

[71] Akenami FO Siren V Koskiniemi M Siimes MA Teravainen H Vaheri A. Cerebrospinal fluid activity of tissue plasminogen activator in patients with neurological diseases. J Clin Pathol (1996) 49(7):577–80. 10.1136/jcp.49.7.577 8813958 PMC500574

[72] Mazodier K Arnaud L Mathian A Costedoat-Chalumeau N Haroche J Frances C Lupus anticoagulant-hypoprothrombinemia syndrome: report of 8 cases and review of the literature. Medicine (Baltimore) (2012) 91(5):251–60. 10.1097/MD.0b013e31826b971f 22932789

[73] Lancellotti S Basso M De Cristofaro R. Congenital prothrombin deficiency: an update. Semin Thromb Hemost (2013) 39(6):596–606. 10.1055/s-0033-1348948 23852823

[74] Hirano K Hirano M. Current perspective on the role of the thrombin receptor in cerebral vasospasm after subarachnoid hemorrhage. J Pharmacol Sci (2010) 114(2):127–33. 10.1254/jphs.10R03CP 20859063

[75] Beilin O Karussis DM Korczyn AD Gurwitz D Aronovich R Hantai D Increased thrombin inhibition in experimental autoimmune encephalomyelitis. J Neurosci Res (2005) 79(3):351–9. 10.1002/jnr.20270 15605378

[76] Lee DM Weinblatt ME. Rheumatoid arthritis. Lancet (2001) 358(9285):903–11. 10.1016/S0140-6736(01)06075-5 11567728

[77] So AK Varisco PA Kemkes-Matthes B Herkenne-Morard C Chobaz-Peclat V Gerster JC Arthritis is linked to local and systemic activation of coagulation and fibrinolysis pathways. J Thromb Haemost (2003) 1(12):2510–5. 10.1111/j.1538-7836.2003.00462.x 14675085

[78] Ingegnoli F Fantini F Favalli EG Soldi A Griffini S Galbiati V Inflammatory and prothrombotic biomarkers in patients with rheumatoid arthritis: effects of tumor necrosis factor-alpha blockade. J Autoimmun (2008) 31(2):175–9. 10.1016/j.jaut.2008.07.002 18707846

[79] Liu X Piela-Smith TH. Fibrin(ogen)-induced expression of ICAM-1 and chemokines in human synovial fibroblasts. J Immunol (2000) 165(9):5255–61. 10.4049/jimmunol.165.9.5255 11046059

[80] Varisco PA Peclat V van Ness K Bischof-Delaloye A So A Busso N. Effect of thrombin inhibition on synovial inflammation in antigen induced arthritis. Ann Rheum Dis (2000) 59(10):781–7. 10.1136/ard.59.10.781 11005778 PMC1753002

[81] Marty I Peclat V Kirdaite G Salvi R So A Busso N. Amelioration of collagen-induced arthritis by thrombin inhibition. J Clin Invest (2001) 107(5):631–40. 10.1172/JCI11064 11238564 PMC199423

[82] Kwiecinski J Josefsson E Jin T. Fibrinolysis is down-regulated in mouse collagen-induced arthritis, but its normalization does not alleviate the course of disease. Inflamm Res (2011) 60(11):1021–9. 10.1007/s00011-011-0363-0 21786185

[83] Xie Z Dai J Yang A Wu Y. A role for bradykinin in the development of anti-collagen antibody-induced arthritis. Rheumatology (Oxford) (2014) 53(7):1301–6. 10.1093/rheumatology/keu015 24599920 PMC4065007

[84] Espinola RG Uknis A Sainz IM Isordia-Salas I Pixley R DeLa Cadena R A monoclonal antibody to high-molecular weight kininogen is therapeutic in a rodent model of reactive arthritis. Am J Pathol (2004) 165(3):969–76. 10.1016/S0002-9440(10)63358-5 15331420 PMC1618603

[85] Keith JC Jr Sainz IM Isordia-Salas I Pixley RA Leathurby Y Albert LM A monoclonal antibody against kininogen reduces inflammation in the HLA-B27 transgenic rat. Arthritis Res Ther (2005) 7(4):R769–76. 10.1186/ar1728 15987478 PMC1175023

[86] Sainz IM Isordia-Salas I Castaneda JL Agelan A Liu B DeLa Cadena RA Modulation of inflammation by kininogen deficiency in a rat model of inflammatory arthritis. Arthritis Rheum (2005) 52(8):2549–52. 10.1002/art.21202 16059911

[87] Valenti C Giuliani S Cialdai C Tramontana M Maggi CA. Anti-inflammatory synergy of MEN16132, a kinin B(2) receptor antagonist, and dexamethasone in carrageenan-induced knee joint arthritis in rats. Br J Pharmacol (2010) 161(7):1616–27. 10.1111/j.1476-5381.2010.00995.x 20726984 PMC3010571

[88] Dela Cadena RA Stadnicki A Uknis AB Sartor RB Kettner CA Adam A Inhibition of plasma kallikrein prevents peptidoglycan-induced arthritis in the Lewis rat. FASEB J (1995) 9(5):446–52. 10.1096/fasebj.9.5.7896018 7896018

[89] Fujimori Y Nakamura T Shimizu K Yamamuro T Wanaka K Okamoto S Effects of a highly selective plasma kallikrein inhibitor on collagen-induced arthritis in mice. Agents Actions (1993) 39(1–2):42–8. 10.1007/BF01975713 8285139

[90] Busso N Peclat V Van Ness K Kolodziesczyk E Degen J Bugge T Exacerbation of antigen-induced arthritis in urokinase-deficient mice. J Clin Invest (1998) 102(1):41–50. 10.1172/JCI2312 9649555 PMC509063

[91] Li J Ny A Leonardsson G Nandakumar KS Holmdahl R Ny T The plasminogen activator/plasmin system is essential for development of the joint inflammatory phase of collagen type II-induced arthritis. Am J Pathol (2005) 166(3):783–92. 10.1016/S0002-9440(10)62299-7 15743790 PMC1602367

[92] Li J Guo Y Holmdahl R Ny T Contrasting roles of plasminogen deficiency in different rheumatoid arthritis models. Arthritis Rheum (2005) 52(8):2541–8. 10.1002/art.21229 16052596

[93] Yang YH Carmeliet P Hamilton JA. Tissue-type plasminogen activator deficiency exacerbates arthritis. J Immunol (2001) 167(2):1047–52. 10.4049/jimmunol.167.2.1047 11441114

[94] Cook AD Braine EL Campbell IK Hamilton JA. Differing roles for urokinase and tissue-type plasminogen activator in collagen-induced arthritis. Am J Pathol (2002) 160(3):917–26. 10.1016/S0002-9440(10)64914-0 11891190 PMC1867189

[95] Cook AD De Nardo CM Braine EL Turner AL Vlahos R Way KJ Urokinase-type plasminogen activator and arthritis progression: role in systemic disease with immune complex involvement. Arthritis Res Ther (2010) 12(2):R37. 10.1186/ar2946 20196869 PMC2888184

[96] De Nardo CM Lenzo JC Pobjoy J Hamilton JA Cook AD. Urokinase-type plasminogen activator and arthritis progression: contrasting roles in systemic and monoarticular arthritis models. Arthritis Res Ther (2010) 12(5):R199. 10.1186/ar3171 20973954 PMC2991036

[97] McLaren M Alkaabi J Connacher M Belch JJ Valenete E. Activated factor XII in rheumatoid arthritis. Rheumatol Int (2002) 22(5):182–4. 10.1007/s00296-002-0219-6 12215862

[98] Uknis AB DeLa Cadena RA Janardham R Sartor RB Whalley ET Colman RW. Bradykinin receptor antagonists type 2 attenuate the inflammatory changes in peptidoglycan-induced acute arthritis in the Lewis rat. Inflamm Res (2001) 50(3):149–55. 10.1007/s000110050739 11339503

[99] Devani M Cugno M Vecchi M Ferrero S Di Berardino F Avesani EC Kallikrein-kinin system activation in Crohn’s disease: differences in intestinal and systemic markers. Am J Gastroenterol (2002) 97(8):2026–32. 10.1111/j.1572-0241.2002.05919.x 12190172

[100] Dolapcioglu C Soylu A Kendir T Ince AT Dolapcioglu H Purisa S Coagulation parameters in inflammatory bowel disease. Int J Clin Exp Med (2014) 7(5):1442–8. 24995109 PMC4073770

[101] Xie J Itzkowitz SH. Cancer in inflammatory bowel disease. World J Gastroenterol (2008) 14(3):378–89. 10.3748/wjg.14.378 18200660 PMC2679126

